# Nine genera of Eucnemidae (Coleoptera) new to Peru, with a key to Peruvian genera

**DOI:** 10.3897/BDJ.3.e4493

**Published:** 2015-03-11

**Authors:** Varpu Vahtera, Jyrki Muona, Ari Linna, Ilari E. Sääksjärvi

**Affiliations:** ‡ Zoological Museum, University of Turku, Turku, Finland; § Zoology Unit, Finnish Museum of Natural History, University of Helsinki, Finland

**Keywords:** Neotropics, Amazon, Elateroidea, eucnemid, lowland, rain forest, white-sand forest, taxonomy, species richness.

## Abstract

Thirteen genera of Eucnemidae containing forty species were collected from the Iquitos region in Peru. Nine of the genera are new to the country: *Rhagomicrus* Fleutiaux, 1902, *Adelorhagus* Horn, 1890, *Adelothyreus* Chevrolat, 1867, *Microrhagus* Dejean, 1833, *Dyscharachthis* Blackburn, 1900, *Heterotaxis* Bonvouloir, 1871, *Spinifornax* Fleutiaux, 1926, *Serrifornax* Fleutiaux, 1926 and *Maelodrus* Fleutiaux, 1928. The previous eucnemid record from Peru contained eleven species in ten genera. Only one of the forty species caught, *Entomophthalmus americanus* Bonvouloir, was previously known and described from the country. *Dyscharachthis*, *Maelodrus* and *Adelorhagus* are recorded from South America for the first time. Many of the collected species seem to favor white-sand forest as their habitat. Possible reasons for this are discussed. A list of eucnemids from Peru is included, containing taxa already recorded from the country and also taxa that are likely to occur there. A key to the Peruvian genera is included.

## Introduction

Eucnemidae is a species rich (185 genera, 1700 species) mainly tropical beetle family, characterized by numerous undescribed species. Studies investigating the abundance of eucnemid beetles are rare, but the few that exist conclude that the family forms a significant portion of the beetle biodiversity in tropical forests (Hammond 1990, Penny and Arias 1982). The evolutionarily most primitive eucnemid groups live in soil as larvae, but all derived groups spend their larval time inside wood. Of these lignicolous groups only a few prefer conifers, the rest live in broad-leaved trees that are infested with white-rot. Despite their larvae living several years in a strictly lignicolous environment, eucnemids are not xylophagous per se. Instead, the few available studies investigating their gut content have shown that the larvae feed on saprotrophic fungus, not on wood (Ford and Spilman 1979, Muona 1993). Adult eucnemids feed very little, if at all, during their short lives.

As is commonly the case in locations with a high diversity, the eucnemid fauna of Peru is still poorly known. Previously, only eleven species belonging to ten genera were reported from the country. In this study we investigate the diversity of eucnemid beetles in Peru as well as discuss the effect that forests growing on white-sand have on the diversity of the group.

### White-sand and clay soil forests in the Peruvian Amazon

The main non-inundated lowland rain forest types in the Peruvian Amazon can be roughly divided into two groups based on the soil they grow on. “Traditional rain forests” are normally forests growing on clayey soil characterized by large trees and vines that form a shady and moist habitat for a rich flora and fauna. In contrast, forests growing on white quartz sand form nutrient-poor habitats that are not preferred by most animals because of their harshness. These forests are called varillal and chamizal in Peru (Encarnación 1985, Ruokolainen and Tuomisto 1993, and caatinga, campina and campinarana in other parts of the Amazon (see Anderson 1981).

Large white-sand areas are known to occur in tropical Asia, Guyana and Brazil (Anderson 1981, MacKinnon et al. 1997) whereas in northern Peru they occur in small isolated patches, surrounded by the prevailing non-inundated rain forests growing on relatively nutrient-rich clayey ground (Räsänen et al. 1998b, Ruokolainen and Tuomisto 1993, Ruokolainen and Tuomisto 1998). In Peru, this unique white-sand forest type is characterized by slender trees and a sparse canopy and shrub layer, typically growing on small hills. In comparison to the generally dry, hot and nutrient-poor white-sand habitats, the shady and moist forests on nutrient-rich clay ground would seem like an ideal habitat for most animals. Indeed, despite several endemic and highly specialized species being reported from white-sand forests (e.g. Alvarez and Whitney 2001), the overall species richness of this habitat type has generally been considered low (MacKinnon et al. 1997, Ruokolainen and Tuomisto 1998). White-sand sites are distributed as isolated patches in the western Amazon. Their quartz-sands were formed from the Sub-Andean foreland in situ weathered sediments by aquatic recycling, sorting and re-deposition. The humid tropical climate speeded up weathering, and the Andean orogeny developing eastwards during the Neogene (25 Ma-recent) was a dynamo creating laterally migrating rivers in the Amazonian lowlands. Floodplains of different age were formed along the sequential uplift of the Andes. Minor rivers and creeks finalized the landscape to consist nowadays of sandy terrains and hills overlying the more resistant, clayish Miocene sediments, which in places forms the forest ground. White sands present the ultimate residual parts of this system, exposed as floodplains of different (depositional) age indicated by their different height, degree of denudation and a minor difference in their maturity (95-99% quartz) (Räsänen et al. 1987, Räsänen et al. 1992, Räsänen et al. 1990, Räsänen et al. 1998a).

## Materials and methods

### Study site and collecting methods

The study was conducted in 1998 and 2000 in the National Reserve of Allpahuayo Mishana (NRAM, 3°57'S, 73°26'W), near the densely populated city of Iquitos (Department of Loreto, Peru). NRAM is famous for its high tropical rain forest habitat heterogeneity, high levels of endemism and extreme species richness (Gentry 1988, Sääksjärvi et al. 2004, Vásquez Martínez and Phillips 2000, Whitney and Alvarez 1998). The soil of NRAM consists of a mosaic of patches varying from white-sand (Anderson 1981, Encarnación 1985) to clay, reflecting the complex geological history and formations of the surface (Räsänen et al. 1998b).

Sampling was conducted using Malaise traps in five areas containing similar kinds of non-inundated rain forest types (see Sääksjärvi et al. 2004, Sääksjärvi et al. 2006). The main aim of these field studies was to sample parasitoid wasps (see Sääksjärvi et al. 2004). In each area two traps were placed in forest growing on clayey to loamy ground (high to intermediate in nutrients) and three traps in forest patches growing on nutrient-poor white-sand soils of differing structure (representing the diversity of white-sand forests present in NRAM) in order to assure that all traps functioned independently. The resulting material was used in the current study since Malaise traps have proved efficient in collecting eucnemid beetles (Hammond 1990). The traps were emptied every second week and the specimens were preserved in 75% alcohol.

Specimens were identified by JM. Part of the collected and identified material will be delivered to the Museum of Natural History, University of San Marcos, Lima, Peru where it will form part of the reference collection on Peruvian eucnemids. The rest of the material is deposited at the Finnish Museum of Natural History, Finland, where it is curated by JM. The new species will be described in connection of generic revisions of global scope.

## Taxon treatments

### Adelorhagus sp. 1

#### Materials

- **Type status:** Other material. **Occurrence:** individualCount: 2; **Taxon:** higherClassification: Coleoptera; Eucnemidae; Melasinae; Dirhagini; genus: Adelorhagus Horn, 1890; **Location:** continent: South America; country: Peru; county: Loreto; municipality: Iquitos; locality: National Reserve of Allpahuayo Mishana (NRAM)

#### Notes

One undescribed species was recorded from clay soil forest (Suppl. material 2). This is the first record of this genus from Peru and South America.

### Adelothyreus sp. 1

#### Materials

- **Type status:** Other material. **Occurrence:** individualCount: 1; **Taxon:** higherClassification: Coleoptera; Eucnemidae; Melasinae; Dirhagini; genus: Adelothyreus Chevrolat, 1867; **Location:** continent: South America; country: Peru; county: Loreto; municipality: Iquitos; locality: National Reserve of Allpahuayo Mishana (NRAM)

#### Notes

An undescribed species was caught in white-sand forest (Suppl. material 2).

### Entomophthalmus americanus

Bonvouloir, 1972

#### Materials

- **Type status:** Other material. **Occurrence:** individualCount: 38; **Taxon:** higherClassification: Coleoptera; Eucnemidae; Melasinae; Dirhagini; genus: Entomophthalmus Bonvouloir, 1871; **Location:** continent: South America; country: Peru; county: Loreto; municipality: Iquitos; locality: National Reserve of Allpahuayo Mishana (NRAM)

#### Notes

This is the only species found in our study that was previously known from Peru (Schenkling 1928). It was widespread and common in our material, present in 15 sites in both clay and white-soil forest (Suppl. material 2).

### Entomophthalmus sp. 1

#### Materials

- **Type status:** Other material. **Occurrence:** individualCount: 2; **Taxon:** higherClassification: Coleoptera; Eucnemidae; Melasinae; Dirhagini; genus: Entomophthalmus Bonvouloir, 1871; **Location:** continent: South America; country: Peru; county: Loreto; municipality: Iquitos; locality: National Reserve of Allpahuayo Mishana (NRAM)

#### Notes

An undescribed species represented by two individuals was found in both forest types (Suppl. material 2).

### Microrhagus sp. 1

#### Materials

- **Type status:** Other material. **Occurrence:** individualCount: 2; **Taxon:** higherClassification: Coleoptera; Eucnemidae; Melasinae; Dirhagini; genus: Microrhagus Dejean, 1833; **Location:** continent: South America; country: Peru; county: Loreto; municipality: Iquitos; locality: National Reserve of Allpahuayo Mishana (NRAM)

#### Notes

An undescribed species found in both forest types (Suppl. material 2).

### Microrhagus sp. 2

#### Materials

- **Type status:** Other material. **Occurrence:** individualCount: 5; **Taxon:** higherClassification: Coleoptera; Eucnemidae; Melasinae; Dirhagini; genus: Microrhagus Dejean, 1833; **Location:** continent: South America; country: Peru; county: Loreto; municipality: Iquitos; locality: National Reserve of Allpahuayo Mishana (NRAM)

#### Notes

An undescribed species found from both forest types (Suppl. material 2).

### Microrhagus sp. 3

#### Materials

- **Type status:** Other material. **Occurrence:** individualCount: 3; **Taxon:** higherClassification: Coleoptera; Eucnemidae; Melasinae; Dirhagini; genus: Microrhagus Dejean, 1833; **Location:** continent: South America; country: Peru; county: Loreto; municipality: Iquitos; locality: National Reserve of Allpahuayo Mishana (NRAM)

#### Notes

An undescribed species found from both forest types (Suppl. material 2).

### Microrohagus sp. 4

#### Materials

- **Type status:** Other material. **Occurrence:** individualCount: 2; **Taxon:** higherClassification: Coleoptera; Eucnemidae; Melasinae; Dirhagini; genus: Microrhagus Dejean, 1833; **Location:** continent: South America; country: Peru; county: Loreto; municipality: Iquitos; locality: National Reserve of Allpahuayo Mishana (NRAM)

#### Notes

An undescribed species found from white-sand forest (Suppl. material 2).

### Microrhagus sp. 5

#### Materials

- **Type status:** Other material. **Occurrence:** individualCount: 4; **Taxon:** higherClassification: Coleoptera; Eucnemidae; Melasinae; Dirhagini; genus: Microrhagus Dejean, 1833; **Location:** continent: South America; country: Peru; county: Loreto; municipality: Iquitos; locality: National Reserve of Allpahuayo Mishana (NRAM)

#### Notes

An undescribed species found from white-sand forest (Suppl. material 2).

### Microrhagus sp. 6

#### Materials

- **Type status:** Other material. **Occurrence:** individualCount: 2; **Taxon:** higherClassification: Coleoptera; Eucnemidae; Melasinae; Dirhagini; genus: Microrhagus Dejean, 1833; **Location:** continent: South America; country: Peru; county: Loreto; municipality: Iquitos; locality: National Reserve of Allpahuayo Mishana (NRAM)

#### Notes

An undescribed species found from white-sand forest (Suppl. material 2).

### Microrhagus sp. 7

#### Materials

- **Type status:** Other material. **Occurrence:** individualCount: 1; **Taxon:** higherClassification: Coleoptera; Eucnemidae; Melasinae; Dirhagini; genus: Microrhagus Dejean, 1833; **Location:** continent: South America; country: Peru; county: Loreto; municipality: Iquitos; locality: National Reserve of Allpahuayo Mishana (NRAM)

#### Notes

An undescribed species was found from white-sand forest (Suppl. material 2).

### Rhagomicrus sp. 1

#### Materials

- **Type status:** Other material. **Occurrence:** individualCount: 2; **Taxon:** higherClassification: Coleoptera; Eucnemidae; Melasinae; Dirhagini; genus: Rhagomicrus Fleutiaux, 1902; **Location:** continent: South America; country: Peru; county: Loreto; municipality: Iquitos; locality: National Reserve of Allpahuayo Mishana (NRAM)

#### Notes

The first record of this genus from Peru. One undescribed species was caught in both forest types (Suppl. material 2).

### Weyrauchiella

Cobos, 1972

#### Materials

- **Type status:** Other material. **Taxon:** higherClassification: Coleoptera; Eucnemidae; Melasinae; Dirhagini; genus: Weyrauchiella Cobos, 1972

#### Notes

*Weyrauchiella peruviana* Cobos, 1972 was described from Tingo Maria, Rio Huallaga, a limestone mountain range area in Peru (Cobos 1972). Additional records from the Andean region are known to us, but this species was not found in our study.

### Dyscharachthis sp. 1

#### Materials

- **Type status:** Other material. **Occurrence:** individualCount: 2; **Taxon:** higherClassification: Coleoptera; Eucnemidae; Eucneminae; Dyscharachthini; genus: Dyscharachthis Blackburn, 1900; **Location:** continent: South America; country: Peru; county: Loreto; municipality: Iquitos; locality: National Reserve of Allpahuayo Mishana (NRAM)

#### Notes

An undescribed species was caught in a white-sand site (Suppl. material 2).

### Idiotarsus sp. 1

#### Materials

- **Type status:** Other material. **Occurrence:** individualCount: 5; **Taxon:** higherClassification: Coleoptera; Eucnemidae; Eucneminae; Eucnemini; genus: Idiotarsus Bonvouloir, 1871; **Location:** continent: South America; country: Peru; county: Loreto; municipality: Iquitos; locality: National Reserve of Allpahuayo Mishana (NRAM)

#### Notes

An undescribed species found in white-sand forest (Suppl. material 2).

### Idiotarsus sp. 2

#### Materials

- **Type status:** Other material. **Occurrence:** individualCount: 13; **Taxon:** higherClassification: Coleoptera; Eucnemidae; Eucneminae; Eucnemini; genus: Idiotarsus Bonvouloir, 1871; **Location:** continent: South America; country: Peru; county: Loreto; municipality: Iquitos; locality: National Reserve of Allpahuayo Mishana (NRAM)

#### Notes

An undescribed species was caught in both forest types (Suppl. material 2). Another undescribed *Idiotarsus* species is previously known from peru (JM collection).

### Ceratogonys

Perty, 1830

#### Materials

- **Type status:** Other material. **Taxon:** higherClassification: Coleoptera; Eucnemidae; Macraulacinae; Orodotini; genus: Ceratogonys Perty, 1830

#### Notes

The genus was not found in our study. Previously one species, *Ceratogonys spinicornis* Fabricius, 1801, is reported from Peru (Schenkling 1928).

### Dromaeolus sp. 1

#### Materials

- **Type status:** Other material. **Occurrence:** individualCount: 2; **Taxon:** higherClassification: Coleoptera; Eucnemidae; Macraulacinae; Macraulacini; genus: Dromaeolus Kiesenwetter, 1858; **Location:** continent: South America; country: Peru; county: Loreto; municipality: Iquitos; locality: National Reserve of Allpahuayo Mishana (NRAM)

#### Notes

An undescribed species was found from both forest types (Suppl. material 2).

### Dromaeolus sp. 2

#### Materials

- **Type status:** Other material. **Occurrence:** individualCount: 4; **Taxon:** higherClassification: Coleoptera; Eucnemidae; Macraulacinae; Macraulacini; genus: Dromaeolus Kiesenwetter, 1858; **Location:** continent: South America; country: Peru; county: Loreto; municipality: Iquitos; locality: National Reserve of Allpahuayo Mishana (NRAM)

#### Notes

An undescribed species was caught in white-sand forest (Suppl. material 2).

### Dromaeolus sp. 3

#### Materials

- **Type status:** Other material. **Occurrence:** individualCount: 2; **Taxon:** higherClassification: Coleoptera; Eucnemidae; Macraulacinae; Macraulacini; genus: Dromaeolus Kiesenwetter, 1858; **Location:** continent: South America; country: Peru; county: Loreto; municipality: Iquitos; locality: National Reserve of Allpahuayo Mishana (NRAM)

#### Notes

An undescribed species was caught in clayey forest (Suppl. material 2).

### Dromaeolus sp. 4

#### Materials

- **Type status:** Other material. **Occurrence:** individualCount: 1; **Taxon:** higherClassification: Coleoptera; Eucnemidae; Macraulacinae; Macraulacini; genus: Dromaeolus Kiesenwetter, 1858; **Location:** continent: South America; country: Peru; county: Loreto; municipality: Iquitos; locality: National Reserve of Allpahuayo Mishana (NRAM)

#### Notes

An undescribed species found in a white-sand site (Suppl. material 2).

### Dromaeolus sp. 5

#### Materials

- **Type status:** Other material. **Occurrence:** individualCount: 1; **Taxon:** higherClassification: Coleoptera; Eucnemidae; Macraulacinae; Macraulacini; genus: Dromaeolus Kiesenwetter, 1858; **Location:** continent: South America; country: Peru; county: Loreto; municipality: Iquitos; locality: National Reserve of Allpahuayo Mishana (NRAM)

#### Notes

An undescribed species was found in a white-sand site (Suppl. material 2).

### Dromaeolus sp. 6

#### Materials

- **Type status:** Other material. **Occurrence:** individualCount: 8; **Taxon:** higherClassification: Coleoptera; Eucnemidae; Macraulacinae; Macraulacini; genus: Dromaeolus Kiesenwetter, 1858; **Location:** continent: South America; country: Peru; county: Loreto; municipality: Iquitos; locality: National Reserve of Allpahuayo Mishana (NRAM)

#### Notes

An undescribed species caught in both forest types (Suppl. material 2).

### Dromaeolus sp. 7

#### Materials

- **Type status:** Other material. **Occurrence:** individualCount: 1; **Taxon:** higherClassification: Coleoptera; Eucnemidae; Macraulacinae; Macraulacini; genus: Dromaeolus Kiesenwetter, 1858; **Location:** continent: South America; country: Peru; county: Loreto; municipality: Iquitos; locality: National Reserve of Allpahuayo Mishana (NRAM)

#### Notes

An undescribed species was caught in a clayey forest site (Suppl. material 2).

### Dromaeolus sp. 8

#### Materials

- **Type status:** Other material. **Occurrence:** individualCount: 1; **Taxon:** higherClassification: Coleoptera; Eucnemidae; Macraulacinae; Macraulacini; genus: Dromaeolus Kiesenwetter, 1858; **Location:** continent: South America; country: Peru; county: Loreto; municipality: Iquitos; locality: National Reserve of Allpahuayo Mishana (NRAM)

#### Notes

An undescribed species caught in a white-sand site (Suppl. material 2).

### Dromaeolus sp. 9

#### Materials

- **Type status:** Other material. **Occurrence:** individualCount: 1; **Taxon:** higherClassification: Coleoptera; Eucnemidae; Macraulacinae; Macraulacini; genus: Dromaeolus Kiesenwetter, 1858; **Location:** continent: South America; country: Peru; county: Loreto; municipality: Iquitos; locality: National Reserve of Allpahuayo Mishana (NRAM)

#### Notes

An undescribed species caught in a clayey forest site (Suppl. material 2). Previously one species, *Dromaeolus morio* (Erichson 1847), was known from Peru (Schenkling 1928).

### Fornax sp. 1

#### Materials

- **Type status:** Other material. **Occurrence:** individualCount: 4; **Taxon:** higherClassification: Coleoptera; Eucnemidae; Macraulacinae; Macraulacini; genus: Fornax Laporte, 1835; **Location:** continent: South America; country: Peru; county: Loreto; municipality: Iquitos; locality: National Reserve of Allpahuayo Mishana (NRAM)

#### Notes

An undescribed species was caught in white-sand forest (Suppl. material 2). An undescribed species is previously known from Peru (JM collection).

### Fornax sp. 2

#### Materials

- **Type status:** Other material. **Occurrence:** individualCount: 3; **Taxon:** higherClassification: Coleoptera; Eucnemidae; Macraulacinae; Macraulacini; genus: Fornax Laporte, 1835; **Location:** continent: South America; country: Peru; county: Loreto; municipality: Iquitos; locality: National Reserve of Allpahuayo Mishana (NRAM)

#### Notes

An undescribed species caught in white-sand forest (Suppl. material 2).

### Fornax sp. 3

#### Materials

- **Type status:** Other material. **Occurrence:** individualCount: 5; **Taxon:** higherClassification: Coleoptera; Eucnemidae; Macraulacinae; Macraulacini; genus: Fornax Laporte, 1835; **Location:** continent: South America; country: Peru; county: Loreto; municipality: Iquitos; locality: National Reserve of Allpahuayo Mishana (NRAM)

#### Notes

An undescribed species caught in white-sand forest (Suppl. material 2).

### Fornax sp. 4

#### Materials

- **Type status:** Other material. **Occurrence:** individualCount: 3; **Taxon:** higherClassification: Coleoptera; Eucnemidae; Macraulacinae; Macraulacini; genus: Fornax Laporte, 1835; **Location:** continent: South America; country: Peru; county: Loreto; municipality: Iquitos; locality: National Reserve of Allpahuayo Mishana (NRAM)

#### Notes

An undescribed species caught in both forest types (Suppl. material 2).

### Fornax sp. 5

#### Materials

- **Type status:** Other material. **Occurrence:** individualCount: 2; **Taxon:** higherClassification: Coleoptera; Eucnemidae; Macraulacinae; Macraulacini; genus: Fornax Laporte, 1835; **Location:** continent: South America; country: Peru; county: Loreto; municipality: Iquitos; locality: National Reserve of Allpahuayo Mishana (NRAM)

#### Notes

An undescribed species caught in white-sand forest (Suppl. material 2).

### Fornax sp. 6

#### Materials

- **Type status:** Other material. **Occurrence:** individualCount: 2; **Taxon:** higherClassification: Coleoptera; Eucnemidae; Macraulacinae; Macraulacini; genus: Fornax Laporte, 1835; **Location:** continent: South America; country: Peru; county: Loreto; municipality: Iquitos; locality: National Reserve of Allpahuayo Mishana (NRAM)

#### Notes

An undescribed species was caught in both forest types (Suppl. material 2).

### Fornax sp. 7

#### Materials

- **Type status:** Other material. **Occurrence:** individualCount: 1; **Taxon:** higherClassification: Coleoptera; Eucnemidae; Macraulacinae; Macraulacini; genus: Fornax Laporte, 1835; **Location:** continent: South America; country: Peru; county: Loreto; municipality: Iquitos; locality: National Reserve of Allpahuayo Mishana (NRAM)

#### Notes

An undescribed species was caught in a white-sand site (Suppl. material 2).

### Fornax sp. 8

#### Materials

- **Type status:** Other material. **Occurrence:** individualCount: 1; **Taxon:** higherClassification: Coleoptera; Eucnemidae; Macraulacinae; Macraulacini; genus: Fornax Laporte, 1835; **Location:** continent: South America; country: Peru; county: Loreto; municipality: Iquitos; locality: National Reserve of Allpahuayo Mishana (NRAM)

#### Notes

A single individual of an undescribed species was caught in a white-sand site (Suppl. material 2).

### Fornax sp. 9

#### Materials

- **Type status:** Other material. **Occurrence:** individualCount: 1; **Taxon:** higherClassification: Coleoptera; Eucnemidae; Macraulacinae; Macraulacini; genus: Fornax Laporte, 1835; **Location:** continent: South America; country: Peru; county: Loreto; municipality: Iquitos; locality: National Reserve of Allpahuayo Mishana (NRAM)

#### Notes

A single individual of an undescribed species was caught in a clayey forest site (Suppl. material 2).

### Fornax sp. 10

#### Materials

- **Type status:** Other material. **Occurrence:** individualCount: 1; **Taxon:** higherClassification: Coleoptera; Eucnemidae; Macraulacinae; Macraulacini; genus: Fornax Laporte, 1835; **Location:** continent: South America; country: Peru; county: Loreto; municipality: Iquitos; locality: National Reserve of Allpahuayo Mishana (NRAM)

#### Notes

A single individual of an undescribed species was caught in a clayey forest site (Suppl. material 2).

### Fornax sp. 11

#### Materials

- **Type status:** Other material. **Occurrence:** individualCount: 3; **Taxon:** higherClassification: Coleoptera; Eucnemidae; Macraulacinae; Macraulacini; genus: Fornax Laporte, 1835; **Location:** continent: South America; country: Peru; county: Loreto; municipality: Iquitos; locality: National Reserve of Allpahuayo Mishana (NRAM)

#### Notes

An undescribed species was caught in both forest types (Suppl. material 2).

### Gagatellus

Fleutiaux, 1912

#### Materials

- **Type status:** Other material. **Taxon:** higherClassification: Coleoptera; Eucnemidae; Macraulacinae; Macraulacini; genus: Gagatellus Fleutiaux, 1912

#### Notes

The genus was not found in our study. Preiviously one species, *Gagatellus baeri* Fleutiaux, 1912, is reported from Peru (Schenkling 1928).

### Heterotaxis sp. 1

#### Materials

- **Type status:** Other material. **Occurrence:** individualCount: 1; **Taxon:** higherClassification: Coleoptera; Eucnemidae; Macraulacinae; Macraulacini; genus: Heterotaxis Bonvouloir, 1871; **Location:** continent: South America; country: Peru; county: Loreto; municipality: Iquitos; locality: National Reserve of Allpahuayo Mishana (NRAM)

#### Notes

This is the first record of this genus from Peru. A single individual of an undescribed species was caught in a white-sand site (Suppl. material 2).

### Macraulacus

Bonvouloir, 1871

#### Materials

- **Type status:** Other material. **Taxon:** higherClassification: Coleoptera; Eucnemidae; Macraulacinae; Macraulacini; genus: Macraulacus Bonvouloir, 1871

#### Notes

Not found in our study, but an undescribed species is previously known from Peru (JM collection).

### Maelodrus sp. 1

#### Materials

- **Type status:** Other material. **Occurrence:** individualCount: 1; **Taxon:** higherClassification: Coleoptera; Eucnemidae; Macraulacinae; Macraulacini; genus: Maelodrus Fleutiaux, 1928; **Location:** continent: South America; country: Peru; county: Loreto; municipality: Iquitos; locality: National Reserve of Allpahuayo Mishana (NRAM)

#### Notes

One individual caught in a white-sand site (Suppl. material 2). This genus was formerly known from the Western Pacific and Australian regions. The undescribed species found in our study exhibits all the diagnostic features of the genus: the antennae are slightly flattened, basally with faintly keeled antennomeres, the abdominal tip is deeply excavated, the lateral antennal grooves are somewhat removed from the lateral border of hypomera and become fainter caudad, and the dorsal vestiture is unevenly distributed, forming faint patterns.

### Nematodes

Berthold, 1827

#### Materials

- **Type status:** Other material. **Taxon:** higherClassification: Coleoptera; Eucnemidae; Macraulacinae; Nematodini; genus: Nematodes Berthold, 1827

#### Notes

We did not find this genus in our study. One species (*Nematodes peruvianus* Cobos, 1964) is known from Peru (Cobos 1964).

### Plesiofornax sp. 1

#### Materials

- **Type status:** Other material. **Occurrence:** individualCount: 6; **Taxon:** higherClassification: Coleoptera; Eucnemidae; Macraulacinae; Macraulacini; genus: Plesiofornax Coquerell, 1866; **Location:** continent: South America; country: Peru; county: Loreto; municipality: Iquitos; locality: National Reserve of Allpahuayo Mishana (NRAM)

#### Notes

An undescribed species with individuals caught in both forest types was recorded in our study (Suppl. material 2). Another species, *Plesiofornax peruvianus* Fleutiaux 1934, is previously known from Peru (Fleutiaux 1934).

### Serrifornax sp. 1

#### Materials

- **Type status:** Other material. **Occurrence:** individualCount: 1; **Taxon:** higherClassification: Coleoptera; Eucnemidae; Macraulacinae; Macraulacini; genus: Serrifornax Fleutiaux, 1926; **Location:** continent: South America; country: Peru; county: Loreto; municipality: Iquitos; locality: National Reserve of Allpahuayo Mishana (NRAM)

#### Notes

This is the first record of this genus from Peru. A single individual of an undescribed species was caught in a white-sand site (Suppl. material 2).

### Spinifornax sp. 1

#### Materials

- **Type status:** Other material. **Occurrence:** individualCount: 1; **Taxon:** higherClassification: Coleoptera; Eucnemidae; Macraulacinae; Macraulacini; genus: Spinifornax Fleutiaux, 1926; **Location:** continent: South America; country: Peru; county: Loreto; municipality: Iquitos; locality: National Reserve of Allpahuayo Mishana (NRAM)

#### Notes

This is the first record of this genus from Peru. A single individual of an undescribed species was caught in a white-sand site (Suppl. material 2).

## Identification Keys

### A key to eucnemid genera of Peru

**Table:** 

1	Antennomeres 9-11 elongated, 8 clearly shorter and narrower than 9	2
–	Antennomeres 9-11 not enlarged, 8 about as long and wide as 9	3
2	Antennomeres 9-11 serrate or pectinate in males, females larger than 15 mm	* Phlegon *
–	Antennomeres 9-11 neither serrate nor pectinate, females smaller than 15 mm	*** Ceratogonys ***
3	Hypomera with basally closed lateral antennal grooves forming deep basal pockets for reception of antennae (Fig. 1), male protarsomere 1 without a sex comb	4
–	Hypomera either simple (Fig. 2), or with notosternal antennal grooves (Fig. 3), or with basally open evenly deep lateral antennal grooves (Fig. 4) in which case the male protarsomere 1 has a basal sex comb (Fig. 5)	7
4	Clypeus very wide and short, distance between the antennal insertion points 6-10 times the distance from the lower edge of the antennal insertion point to the edge	* Bossionus *
–	Clypeus much narrower, the width at most 4.5 times the height	5
5	Hypomera with pit-like hairy excretory organs (Fig. 6)	*** Idiotarsus ***
–	Hypomera without such structures	6
6	Head simple, frons and clypeus without keels	* Entomosatopus *
–	Frons and/or clypeus with sharp keels	*** Dyscharachthis ***
7	Lateral pronotal ridge minutely serrate (Fig. 7), hypomera usually with notosternal antennal grooves (Fig. 3), male protarsomere 1 usually with an apical sex comb (Fig. 8)	8
–	Lateral pronotal ridge smooth	15
8	Elytral suture forming a beak before the apex in lateral view (Fig. 9)	* Arrhipis *
–	Elytral apex evenly curved to end in lateral view	9
9	Combined length of antennomeres 2 and 3 less than the length of 4	*** Entomophthalmus ***
–	Combined length of antennomeres 2 and 3 always distinctly greater than the length of 4	10
10	Metacoxal plates approximately parallel-sided (Fig. 10)	11
–	Metacoxal plates distinctly wider close to the insertion point of the trochanter than on the sides (Fig. 11)	14
11	Antennal grooves parallel-sided, always well defined, body parallel-sided, antennae feebly serrate, often elongated	*** Rhagomicrus ***
–	Antennal grooves either entirely absent or widening caudad, poorly delimited	12
12	Antennomeres dentate, body uniformly yellow	*** Adelorhagus ***
–	Antennomeres 4-10 serrate or pectinate, dorsum dark or bicoloured	13
13	Pronotum unusually large compared to the rest of the body, body front-heavy in appearance, pronotum black, elytra sometimes with pale spots	*** Adelothyreus ***
–	Dorsum black, pronotum and elytral longitudinal stripes yellow	*** Weyrauchiella ***
14	Width of the frons between antennal sockets less than half the distance between the eyes, usually distinctly less, body usually black or dark brown, male protarsomere 1 with an apical sex comb	*** Microrhagus ***
–	Width of the frons between antennal sockets at least half the distance between the eyes, usually distinctly more, body evenly yellowish brown, male protarsomere 1 without any spine comb	* Golbachia *
15	Hypomera without medially defined antennal grooves (Fig. 2)	16
–	Hypomera with medially sharply defined basally open lateral antennal grooves (Fig. 4)	21
16	Mandibles slender	* Monrosina *
–	Mandibles stout with a secondary basal tooth	17
17	Meso- and metatibiae without spine combs on lateral surfaces, male protarsomere 1 simple	18
–	Meso- and metatibiae with spine combs, male protarsomere 1 with a basal sex comb (Fig. 5)	19
18	Antennomeres 3-10 deeply serrate or flabellate	* Calyptocerus *
–	Antennomeres 3-10 tubular, neither serrate nor flabellate	* Paraxylophilus *
19	Frons usually conspicuously flattened, antennomeres 6-10 slightly enlarged and flattened, 6 always longer and usually wider than 5, 3-10 not serrate	*** Nematodes ***
–	Frons convex, antennomere 5 usually similar in size to 6, antennomeres 3-10 dentate, serrate or tubular, protibiae with simple apexes	20
20	Dorsum shiny or very shiny, at most densely punctate, brownish	*** Plesiofornax ***
–	Dorsum extremely dull, very densely and strongly rugose, black	*** Gagatellus ***
21	Elytral epipleura grooved, smooth and shiny basally	*** Serrifornax ***
–	Elytral epipleura even, not grooved in front	22
22	Abdominal tip excavated, bifid	*** Maelodrus ***
–	Abdominal tip pointed or rounded	23
23	Antennal grooves large in volume, wider than rest of hypomera	*** Macraulacus ***
–	Antennal grooves much narrower than rest of hypomera (Fig. 4)	24
24	Elytra with sharply marked, punctate striae (Fig. 12) parallel-sided in shape	*** Heterotaxis ***
–	Elytral striae faint, never punctate, elytra rarely parallel-sided in shape	25
25	Claws with basal teeth	*** Fornax ***
–	Claws simple	*** Dromaeolus ***

The genera reported either earlier or in this study are shown in bold. The key also includes genera that are still undiscovered in Peru, but likely to be found there because they are known from the surrounding region (shown in italics only).

## Analysis

Since the traps were placed in two different forest types (three traps in white-sand forest and two in forest growing on clayey soil in each area), the average number of species and individuals that each trap collected was calculated. A species accumulation curve was calculated using EstimateS (Colwell 2013) in order to estimate how efficient our sampling was.

**Figure 13.**
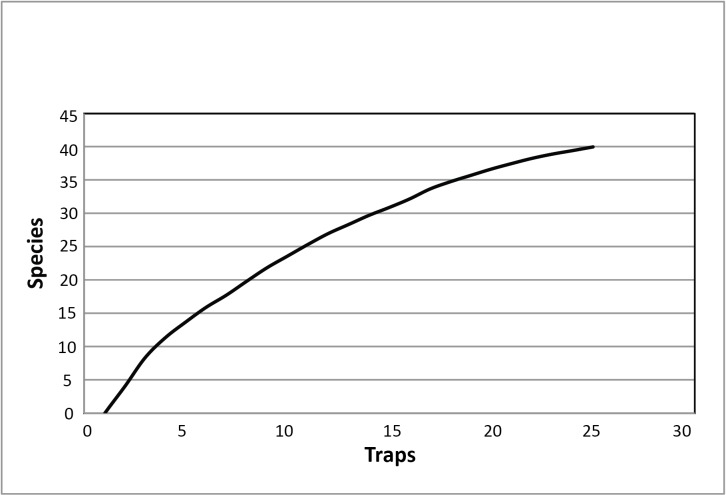
Local species accumulation of eucnemids in Allpahuayo Mishana, Peru (Suppl. material 1).

The total sample size was 185 malaise trap months, which presents one of the largest insect samples ever collected in the western Amazon by Malaise trapping. The material contained 40 eucnemid species belonging to 13 genera; 39 of the species were undescribed. Nine of the collected genera have never been reported from Peru before. Two genera are new to South America as a whole (*Adelorhagus*, *Maelodrus*), and one (*Dyscharachthis*) has only been reported there in passing in a more general context (Muona 1991, Muona 1993). The total number of individuals collected was 141. Average a trap placed in white-sand forest caught 4.5 species and 6.2 individuals whereas a trap in clay soil forest caught 3.8 species and 5.3 individuals. Many of the species were represented by only one or two individuals (the number of singletons and doubletons was 15 and 11, respectively). The species accumulation curve was far from reaching an asymptote indicating that additional sampling would yield a considerable number of new eucnemid species (Fig. 13).

## Discussion

The few studies that have sampled eucnemid diversity (Hammond 1990, Penny and Arias 1982) conclude that the group forms an important component of the beetle biodiversity in tropical forests. Major unpublished material exists from NE Australia, the Fiji Islands and the Grande Terre of New Caledonia. More exact comparisons have to wait for future analyses, but preliminary results suggest that the diversity of the Peruvian fauna is best compared with that of Australia, being somewhat higher than in Fiji and considerably higher than in New Caledonia.

We have shown that by conducting a biodiversity survey in one area in the Peruvian Amazon, we were able to double the number of genera and quadruple the number of species reported from the country. However, despite our sampling being intensive and long-term (185 Malaise trap months in total), it was nowhere near sufficient to record most eucnemid species present in the sampled area. This is indicated by the species accumulation curve showing no sign of stabilizing. Also, the number of rare species remained high throughout the sampling which further indicates the presence of numerous undiscovered species (Colwell and Coddington 1994). The use of other intensive collecting methods, such as light or window trapping, would probably have resulted in a higher species richness (see Longino et al. 2002).

Two of the genera reported here (*Maelodrus* and *Adelothyreus*) have never been collected from South America before: *Adelothyreus* is known from Central America (Costa Rica, unpublished; Panama, *loc. class*.) but the closest reported occurrences of the genus *Maelodrus* are from Western Polynesia and Australia. Furthermore, although the existence of *Dyscharachthis* in South America was briefly noted by Muona (1991), Muona (1993), the genus has not been properly reported from the continent before.

**Figure 14.**
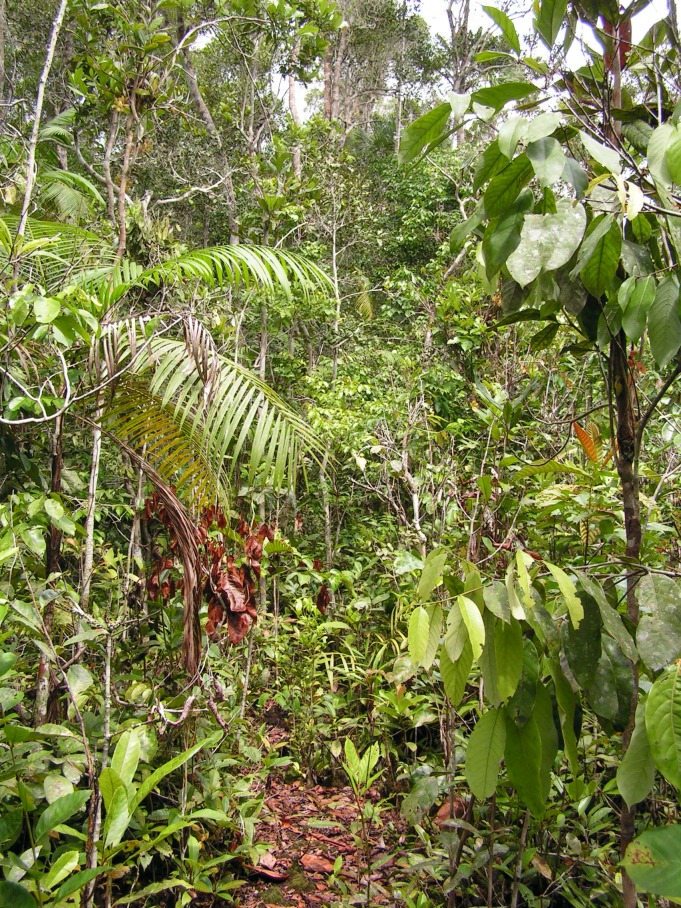
Low canopy white-sand forest (photo: I.E.Sääksjärvi).

**Figure 15.**
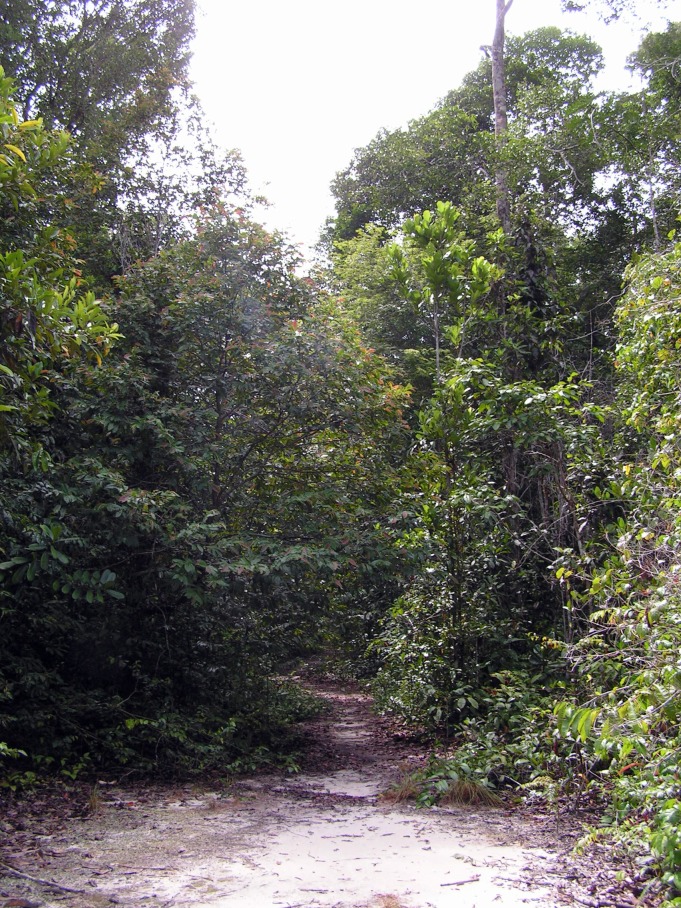
Nutrient-poor white sand exposed in Allpahuayo (photo: I.E.Sääksjärvi).

Many of the new species obtained in this study were caught in study sites located in white-sand forest (see habitats in Figs 14, 15). This is interesting, since for most taxa the overall diversity of this habitat has been considered low (MacKinnon et al. 1997, Ruokolainen and Tuomisto 1993). The main reason that many animal groups avoid white-sand forest as their preferred habitat is its hot, dry and nutrient-poor nature. Evidently there must be something in white-sand forest conditions that attracts and favors eucnemid beetles. Given the complex structure of clay soil forest (e.g. multilayered and tall canopy, abundance of large trees, herbs, vines and epiphytes), one might have expected lignicolous beetles to be richer there than in white-sand forest. White-sand forest is characterized by a rather simple physiognomy (e.g. uniform and rather low canopy height, most tree trunks less than 20 cm in diameter, dominance of a few tree or palm species, absence of large herbs, trees and tree ferns), and a large amount of ectomychorriza (see Anderson 1981). The abundance of saprotrophic fungi could be the key factor attracting eucnemid beetles, as most eucnemid larvae are highly specialized to feed on it (Ford and Spilman 1979, Mamaev 1976, Muona and Teräväinen 2008). The harsh conditions outside the fungi-infested wood may not matter much to these saproxylic beetles that spend most of their life inside the wood.

Another explanation for the high eucnemid diversity in white-sand forest stems from the geological history of the white-sands. White-sand forests in the geologically more stable Central and Eastern Amazon may have been more persistent and extensive than the geologically recently formed and isolated white-sand patches in the western Amazon (Anderson 1981). Although scattered at present, the forests growing on white-sand in the western Amazon may have been open to colonization from more widely distributed white-sand forests in the Central and Eastern Amazon, serving as refugiae for species specialized to this forest type. Moreover, although the species richness in one white-sand patch may be low overall, the heterogeneity of forest types growing on white-sand patches makes the diversity extremely high between different patches. Their differences are regulated by the formation and depth of the impermeable spodic horizon (Klemola 2003) affecting the moisture conditions in the soil. The thickest impermeable horizons have been formed against the Miocene clayish sediments and it is possible that allochthonous material has been enriched in them. Although the quartz-sands are highly nutrient-poor, they occasionally contain small amounts of clay, the capacity of which to release mineral nutrients (Ca^2+^. Mg^2+^, Na^+^ and K^+^) into the system is 2,5 – 5 times less than in the Miocene sediments of the region and five times higher than in the thoroughly weathered clays in the Eastern Amazonia, Guyana Shield (Linna et al. 1998).

Though eucnemid species are mostly characterized by having a relatively small body size, there are also large-sized taxa, of which e.g. the genus *Phlegon* occurs in the Brazilian Amazon. Interestingly, all the species collected in this study are small, 2-8 mm in length. This may just be a matter of low sampling efficiency or alternatively it may reflect the fact that white-sand forest trees are commonly thin (most tree trunks less than 20 cm in diameter). The latter alternative cannot be the sole explanation, however, since clay soil forest also had only small eucnemids despite the presence of large trees.

## Supplementary Material

Supplementary material 1 Species accumulation data Data type: EstimateS results
File: oo_35345.xlsx Vahtera, Muona, Linna, Sääksjärvi

Supplementary material 2 Eucnemid specimens by traps Data type: occurences
Brief description: Data showing the number of specimens / species collected by each trap.
File: oo_36842.xlsx Vahtera, Muona, Linna, Sääksjärvi

XML Treatment for Adelorhagus sp. 1

XML Treatment for Adelothyreus sp. 1

XML Treatment for Entomophthalmus americanus

XML Treatment for Entomophthalmus sp. 1

XML Treatment for Microrhagus sp. 1

XML Treatment for Microrhagus sp. 2

XML Treatment for Microrhagus sp. 3

XML Treatment for Microrohagus sp. 4

XML Treatment for Microrhagus sp. 5

XML Treatment for Microrhagus sp. 6

XML Treatment for Microrhagus sp. 7

XML Treatment for Rhagomicrus sp. 1

XML Treatment for Weyrauchiella

XML Treatment for Dyscharachthis sp. 1

XML Treatment for Idiotarsus sp. 1

XML Treatment for Idiotarsus sp. 2

XML Treatment for Ceratogonys

XML Treatment for Dromaeolus sp. 1

XML Treatment for Dromaeolus sp. 2

XML Treatment for Dromaeolus sp. 3

XML Treatment for Dromaeolus sp. 4

XML Treatment for Dromaeolus sp. 5

XML Treatment for Dromaeolus sp. 6

XML Treatment for Dromaeolus sp. 7

XML Treatment for Dromaeolus sp. 8

XML Treatment for Dromaeolus sp. 9

XML Treatment for Fornax sp. 1

XML Treatment for Fornax sp. 2

XML Treatment for Fornax sp. 3

XML Treatment for Fornax sp. 4

XML Treatment for Fornax sp. 5

XML Treatment for Fornax sp. 6

XML Treatment for Fornax sp. 7

XML Treatment for Fornax sp. 8

XML Treatment for Fornax sp. 9

XML Treatment for Fornax sp. 10

XML Treatment for Fornax sp. 11

XML Treatment for Gagatellus

XML Treatment for Heterotaxis sp. 1

XML Treatment for Macraulacus

XML Treatment for Maelodrus sp. 1

XML Treatment for Nematodes

XML Treatment for Plesiofornax sp. 1

XML Treatment for Serrifornax sp. 1

XML Treatment for Spinifornax sp. 1

## Figures and Tables

**Figure 1.**
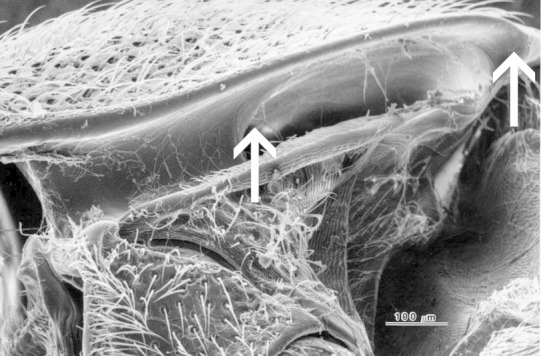
Hypomera with basally closed lateral antennal grooves forming deep basal pockets for reception of antennae.

**Figure 2.**
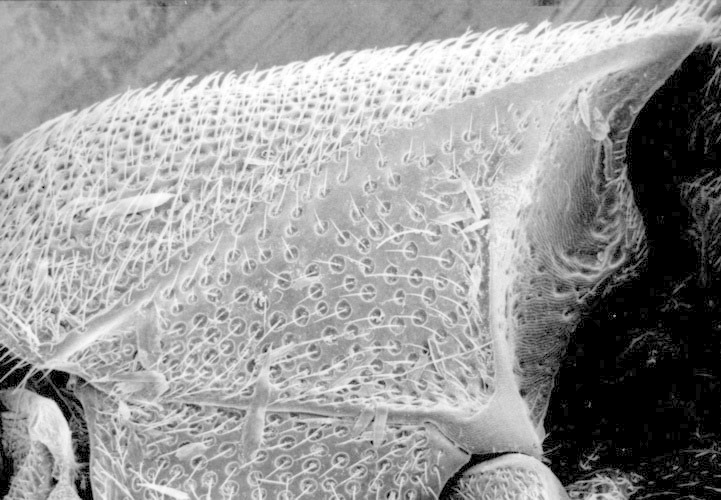
Hypomeron without an antennal groove, Melasinae sp.

**Figure 3.**
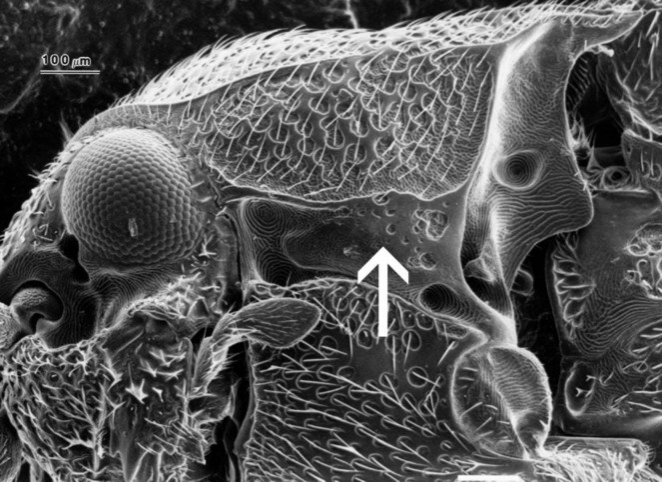
Hypomeron with a notosternal antennal groove, Dirhagini sp.

**Figure 4.**
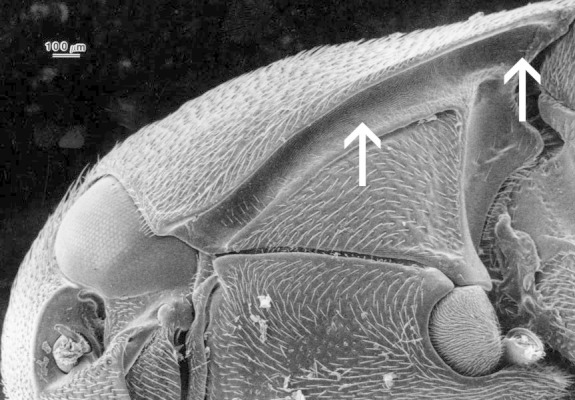
Hypomeron with a lateral antennal groove, Macraulacinae sp.

**Figure 5.**
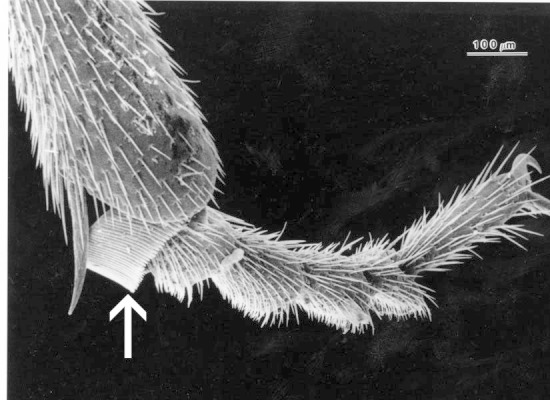
Protarsus with a basal sex comb on tarsomere 1, Macraulacinae sp.

**Figure 6.**
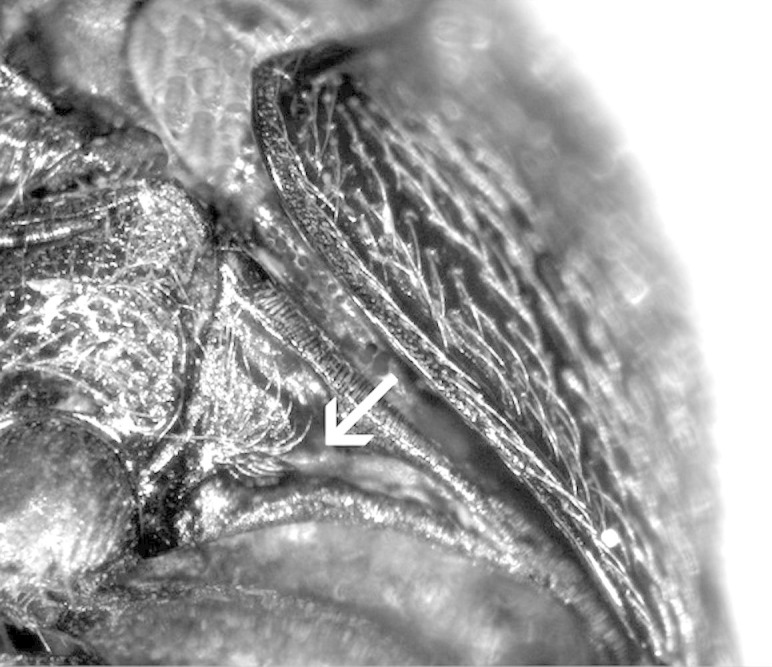
Hypomeron with a sensory pit, Eucnemini sp.

**Figure 7.**
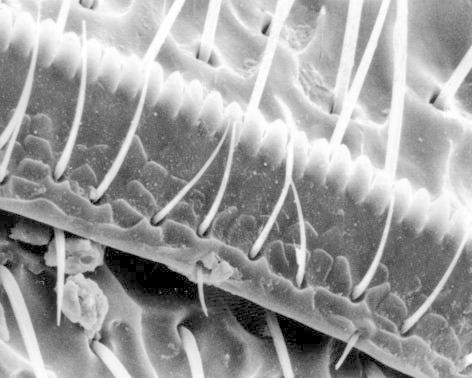
Front edge of pronotum with a serrate ridge, Dirhagini sp.

**Figure 8.**
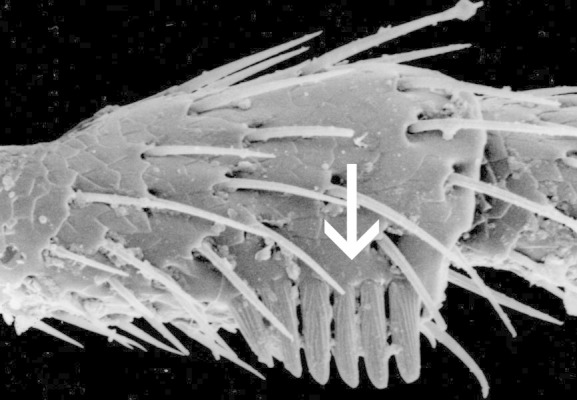
Protarsus with an apical sex comb on tarsomere 1, Dirhagini sp.

**Figure 9.**
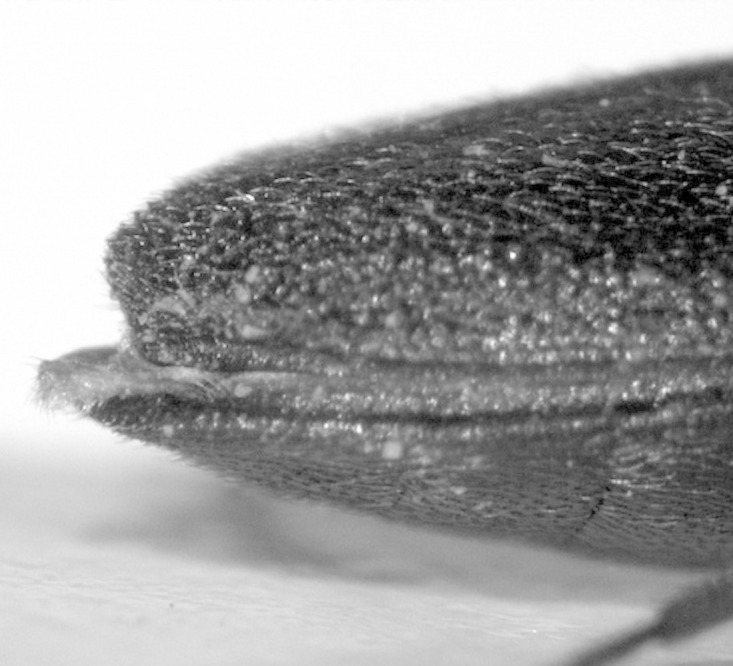
Rounded tip of the elytra, Dirhagini sp.

**Figure 10.**
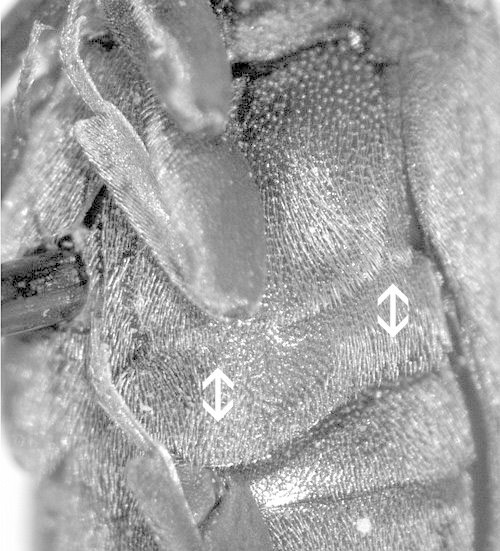
Uniformly wide metacoxal plate, Macraulacinae sp.

**Figure 11.**
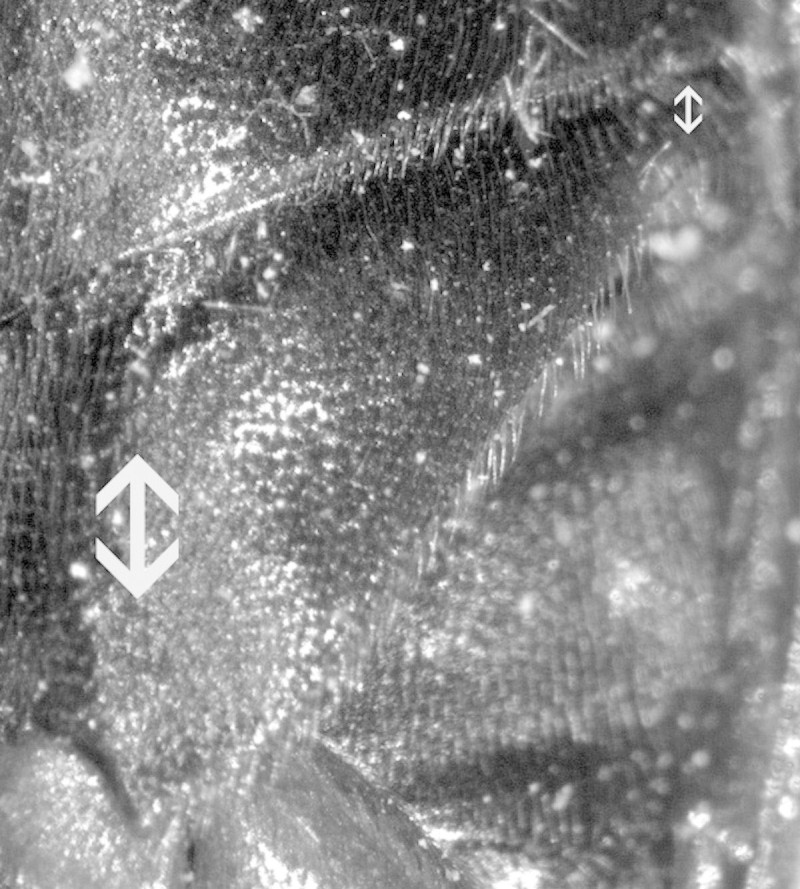
Laterally narrowing metacoxal plate, Macraulacinae sp.

**Figure 12.**
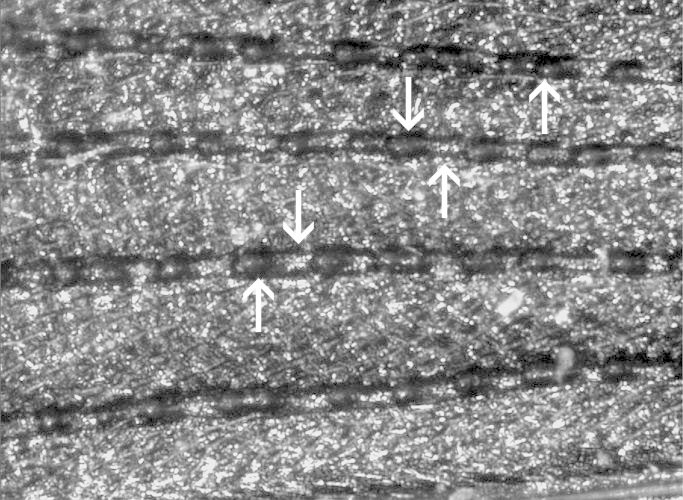
Punctate elytral striae, Macraulacinae sp.
